# Perceived Need for Mental Health Care and Associated Factors and Outcomes in Older Adults Consulting in Primary Care

**DOI:** 10.1177/07067437211055430

**Published:** 2021-11-26

**Authors:** Catherine Lamoureux-Lamarche, Djamal Berbiche, Helen-Maria Vasiliadis

**Affiliations:** 1 Faculty of Medicine and Health Sciences, 12370 Campus de Longueuil—Université de Sherbrooke, Longueuil, Quebec, Canada; 2 Centre de recherche Charles-Le Moyne, 150 Place Charles-Le Moyne, Longueuil, Quebec, Canada

**Keywords:** perceived need, depression, anxiety disorders, factors, health-related outcomes, epidemiological study, older adults

## Abstract

**Objective:**

To assess the individual and health system factors and health-related outcomes associated with perceived need for mental health care in older adults consulting in primary care.

**Method:**

This longitudinal cohort study was conducted among 771 cognitively intact older adults aged ≥65 years recruited in primary care practices in Quebec between 2011 and 2013 and followed 4 years later. Predisposing, enabling and need factors were based on Andersen’s framework on help-seeking behaviors. Health-related outcomes included course of common mental disorders (CMDs), change in quality of life and societal costs. Perceived need for care (PNC) was categorized as no need, met and unmet need. Multinomial regression analyses were conducted to assess the association between study variables and PNC in the overall and the subsample of participants with a CMD at baseline.

**Results:**

As compared with individuals reporting no need, those with an unmet need were more likely to have cognitive decline and lower continuity of care; while those with a met need were more likely to report decreased health-related quality of life. As compared with individuals with an unmet need, those reporting a met need were more likely to report ≥ 3 physical diseases and an incident and persistent CMD, and less likely to show cognitive decline. In participants with a CMD, individuals reporting a met as compared with no need were more likely to be categorized as receiving minimally adequate care and a persistent CMD. Need for care was not associated with societal costs related to health service use.

**Conclusions:**

Overall, physicians should focus on individuals with cognitive impairment and lower continuity of care which was associated with unmet mental health need. Improved follow-up in these populations may improve health care needs and outcomes.

## Abrégé

**Objectif:**

Évaluer les facteurs individuels et du système de santé ainsi que les résultats de santé associés au besoin en soins de santé mentale perçu chez les personnes âgées qui consultent en première ligne.

**Méthode:**

La présente étude de cohorte longitudinale a été menée auprès de 771 adultes âgés ≥ 65 ans, sans problème cognitif, recrutés dans des cliniques de soins de première ligne au Québec entre 2011 et 2013 et suivis quatre ans plus tard. Les facteurs prédisposants, facilitateurs et de besoins étaient basés sur le modèle d’Andersen portant sur les attitudes de recherche d’aide. Les résultats liés à la santé incluaient le patron temporel des troubles mentaux courants (TMC), le changement de la qualité de vie et les coûts sociétaux. Le besoin de soins perçu (BSP) était catégorisé en aucun besoin, besoin comblé et besoin non comblé. Des analyses de régression multinomiale ont été menées pour évaluer l’association entre les variables à l’étude et le BSP dans l’échantillon global et le sous-échantillon des participants présentant un TMC au départ.

**Résultats:**

Comparativement aux personnes ne déclarant aucun besoin, celles ayant un besoin non comblé étaient plus susceptibles de rapporter un déclin cognitif et une continuité de soins plus faible; alors que celles ayant un besoin comblé étaient plus susceptibles de déclarer une réduction de la qualité de vie liée à la santé. Comparativement aux personnes ayant un besoin non comblé, celles qui rapportent un besoin comblé étaient plus susceptibles de déclarer ≥ 3 maladies physiques et un TMC incident et persistant, et moins susceptibles de présenter un déclin cognitif. Chez les participants ayant un TMC, ceux qui rapportent un besoin comblé par rapport à ceux sans aucun besoin sont plus susceptibles d’être catégorisés comme ayant reçu des soins minimallement adéquats et un TMC persistant. Le besoin de soins n’était pas associé aux coûts sociétaux associés à l'utilisation des services de santé.

**Conclusions:**

En général, les médecins devraient mettre l’accent sur les personnes ayant une déclin cognitif et une continuité de soins plus faible, qui était associée aux besoins de santé mentale non comblés. Un meilleur suivi dans ces populations peut améliorer les besoins en soins et les résultats de santé.

## Introduction

The concept of quality of care is multidimensional and includes patient-centered care, security, equity, timely access, efficacy and efficiency.^1^ Perceived need for mental health care has been used to assess patient-centered care in observational studies.^2^ A previous Canadian study showed that 86% and 13% of adults with and without a mental and substance use disorder reported a need for mental health care.^3^ Similar estimates were reported in the Netherlands with 74% of adults with a common mental disorder reporting a need for mental care.^4^ When looking at community-dwelling older adults, 50% and 7% of those with and without a mental disorder reported a need for care.^5^

Few studies have looked at the factors associated with unmet and met mental health needs.^6,7^ Among adults with a perceived need, female gender, younger age, the presence of addiction problems, better cognitive functioning and consulting a lower number of health professionals were associated with unmet versus partially and fully met need for mental health care.^6^ A secondary data analysis of a randomized study in adults with depression showed that those reporting unmet need were more likely to have persistent depression at 18 and 24 months follow-up.^8^ Perceived need for care (PNC) in adults with a mental disorder was also associated with poorer quality of life in a cross-sectional population-based study.^9^ A primary care study of adults with depression and anxiety did not show an association between perceived need for mental health care and costs from the societal perspective.^10^

Using Andersen’s^11^ health care-seeking behavior model, the aim of this study was to assess the predisposing, enabling and need factors associated with perceived need for mental health care in older adults consulting in primary care practices. Our hypothesis was that need, enabling and predisposing factors would be associated with a perceived need for mental health care. To our knowledge, no study has documented the factors associated with perceived need for mental health care in primary care older adults. This is important, as the prevalence of psychiatric and physical comorbidity increases with age^12,13^ and over 70% of older adults consult in primary care settings for their mental health symptoms.^14^ This study will inform on the perceived needs for mental health care of older adults and related health outcomes to prepare health care systems for the future predicted growth of the aging population in Canada.^15^

## Methods

The study was conducted among 771 older adults aged ≥65 years participating in the longitudinal ESA-Services cohort study and followed over a 4-year period. Participants gave consent to link their health survey to their medico-administrative data over the study period. Older adults were recruited in primary care between 2011 and 2013 in one of Quebec’s largest administrative regions and were available for the follow-up of this study, 4 years later (2015–2017).

The methodology of the ESA-Services study has been previously described elsewhere.^16,17^ Briefly, French-speaking older adults aged ≥65 years waiting for a medical appointment with a general practitioner (GP) who were interested to participate in the study were interviewed at home by a trained research assistant using a structured computed assisted questionnaire.^18^ Consent forms were explained and signed at the beginning of the computer-assisted interview. Participants with moderate to severe cognitive decline measured at baseline, using the ESA French-Canadian translation of the Mini-Mental State Examination^19,20^ adapted for home interviews in community living older adults, were excluded from the study. Older adults were also invited to give consent to link their health survey and provincial administrative data from the *Régie de l’Assurance Maladie du Québec* (RAMQ) and *Maintenance et exploitation des données pour l'étude de la clientèle hospitalière* (MED-ECHO) databases during the study period. The RAMQ database included, for administrative purposes, physician fees paid out for medical consultations and acts during emergency department and outpatient visits and inpatient stays and diagnoses. Physicians, however, are not required to register diagnoses to be paid. The RAMQ pharmaceutical database includes, for all outpatient prescriptions dispensed, information on date of delivery, dose and duration, drug costs including pharmacist fee, cost of medication and fees paid out by beneficiaries. MED-ECHO provides information on hospitalizations including admission and discharge diagnoses. The analytic sample came from the 1,642 participants who had accepted the linking of their health survey and administrative data at baseline. The research ethics board of the CIUSSS de l’Estrie-Centre Hospitalier Universitaire de Sherbrooke approved the longitudinal ESA-Services study.

## Measures

### Perceived need for mental health care

Past-year perceived need for mental health care was measured at the follow-up interview using an adapted French version of the PNC Questionnaire.^21,22^ All participants were invited to answer this questionnaire regardless of their answer on depression and anxiety symptoms. This instrument included type and perceived adequacy of help received, and the PNC. The type of help received and needed included: (1) information about mental health problems, treatments or available services; (2) medications; (3) counseling, therapy or help regarding interpersonal relationships; and (4) other types of needs. Based on these, the level of perceived need for mental care was categorized as follows: no need, met need and unmet need.^10,21^ The original scale showed good intra-rater reliability and satisfactory construct validity.^21^ For the purpose of this study, met and unmet need were combined to identify individuals with perceived needs. The description of each level is provided in Table 1 and the questions used to create this variable are available in the Supplemental Material (Table S1).

**Table 1.** Description of the levels of perceived need for mental care.

Level of needs	Description
No need	No help received (Q1) and did not report a perceived need for any types of help (Q3)
Met need	Received at least one type of help (Q1), received as much as need (Q2) and did not report a perceived need for another types of help (Q3)
Perceived need	
Unmet need	Received at least one type of help (Q1), but did not receive as much as needed (Q2) OR Perceived a need for a type of help (Q3)

Other study variables of interest included individual and health system factors, which were selected based on Andersen’s framework^11^ on health service use and patient outcomes. All study variables are detailed in Table S2.

### Need Factors

The presence of a common mental disorder (CMD) was based on the presence of a self-reported or a physician diagnosis of depression or an anxiety disorder. With the exception of the criteria referring to functional impairment, self-reported depression and anxiety in the last 6 months were based on DSM-5 criteria.^23^ The presence of a physician diagnosis of CMD, in the 6 months surrounding the baseline and follow-up, was assessed with the International Classification of Diseases 9th and 10th revisions codes.^24-27^ Based on the presence/absence of CMD at baseline and follow-up, the course of CMD was categorized as follows: no CMD, remitted case, incident case and persistent case.

Other individual need factors measured at follow-up included: the presence of three or more chronic physical diseases, percentage of cognitive decline between baseline and follow-up,^19^ psychological distress and anxiety symptoms assessed with the Kessler Psychological Distress Scale (K10)^28^ and the Seven-item Generalized Anxiety Disorder (GAD-7),^29^ and the number of daily hassles (0–22).^30^

### Enabling Factors

Quality of life included health-related quality of life (HRQOL), measured using a visual anolog scale ranging from 0 to 100, and satisfaction with life (Table S2).^31^ Satisfaction with life was assessed using an adapted French version of the Satisfaction With Life Scale.^32,33^ This measure included five questions on a five-point Likert scale ranging from “completely disagree” to “completely agree” with a total score varying from 5 to 25. The original seven-point Likert scale was reduced to decrease the burden on participants during the interview. The percentage of change between baseline and follow-up was calculated.

Total 3-year costs from the societal perspective included costs from the health system and patient perspectives and were based on guidelines for economic evaluations, published methodologies and the literature.^34-39^ Health care utilization was assessed from the RAMQ and MED-ECHO databases. Unit costs were assessed based on 2013–2014 published financial reports from the Quebec Ministry of health and social services^40,41^ and data from the literature.^34,35,42-45^.

Individual enabling factors also included material and social deprivation indexes of areas of residence,^46^ the presence of social support at follow-up (0–3),^47^ adequacy of care received for CMD at baseline and the Bice–Boxerman continuity of care index^48^ calculated for the 3 years following baseline. The definition of minimally adequate quality of care for pharmacological or psychological treatment received for CMD was based on Canadian clinical guidelines and relevant literature.^49-56^ To be categorized as receiving adequate or inadequate care, participants had to be covered under Quebec’s public drug plan for the 6 months preceding and 9 months following the baseline interview.

Enabling health system factors included type of primary care clinic of recruitment and was categorized as small private clinics with less than three physicians versus family medicine groups, local community health centers and private clinics with at least three physicians. Historical attraction and retention indexes of the area of residence were also considered. The retention indexes measure the proportion of general and psychiatric services consumed by the local population in a region over all services received by this population regardless of the region they received the services. The attraction indexes assess the proportion of general and psychiatric services consumed by the local population in a region over all services rendered in this region.^57^

### Predisposing Factors

Individual predisposing factors included: age, sex (female vs. male), education (primary vs. secondary/post-secondary/university), marital status (married vs. not married) and the presence of adverse childhood experiences.^58^

### Analyses

Descriptive and bivariate analyses were conducted to assess the association between study variables and PNC categorized as (1) no need versus need and (2) no need versus met need versus unmet need. Study variables associated with PNC at a threshold of *p* < 0.10 in the overall sample were included in the multivariable analyses. Multinomial analyses were conducted to study PNC categories as a function of individual and health system factors and health-related outcomes in the overall and subsample of older adults with a CMD at baseline covered under Quebec’s public drug insurance plan. Adequacy of care received for CMD was therefore included in the analyses of the subsample. Odds ratio estimates and 95% CIs are presented with their *p*-value. In the current study sample, 53 older adults had missing information on the material and social deprivation indexes. These variables were imputed using the factors sex and age.^59^ A total of 12 and four participants had missing data on the K10 and GAD-7. For those with at least six out of 10 (K10) and four out of seven (GAD-7) completed items, the mean score of answered questions was used to impute missing data.

## Results

In the overall sample, 67% reported no need for mental health care, while 17% and 16% reported met and unmet needs. In the subsample of older adults with a CMD at baseline, 54% of older adults did not have a PNC, while 27% and 19% reported a met and unmet need at follow-up. Bivariate and multivariate associations for the overall sample and participants with a CMD are presented in Tables 2-4. The association between the presence of a PNC (need vs. no need) and predisposing, enabling and need factors are presented in the Supplemental Material (Table S3).

**Table 2.** Bivariate associations between perceived need for mental health care and study variables

	Overall N=771^a^	No need N=521^a^	Met need N=128^a^	Unmet need N=122^a^	Met need vs no need (ref)		Unmet need vs no need (ref)		Met need vs unmet need (ref)	
	N (%)				Odds ratio	p value	Odds ratio	p value	Odds ratio	p value
*Individual need factors*										
Course of CMD										
No CMD	533 (69.1)	397 (76.2)	60 (46.9)	76 (62.3)	REF		REF		REF	
Incident cases	52 (6.7)	24 (4.6)	17 (13.3)	11 (9.0)	**4.69**	**<0.001**	**2.39**	**0.023**	1.96	0.113
Remission cases	139 (18.0)	85 (16.3)	30 (23.4)	24 (19.7)	**2.34**	**<0.001**	1.48	0.139	1.58	0.156
Persistent cases	47 (6.1)	15 (2.9)	21 (16.4)	11 (9.0)	**9.26**	**<0.001**	**3.83**	**0.001**	**2.42**	**0.031**
Number of chronic physical diseases										
<3 physical disorders	245 (32)	185 (36)	22 (17)	38 (31)	REF		REF		REF	
≥3 physical disorders	526 (68)	336 (64)	106 (83)	84 (69)	**2.65**	**<0.001**	1.22	0.363	**2.18**	**0.011**
	Mean (SD)	Mean (SD)	Mean (SD)	Mean (SD)						
% cognitive decline	0.72 (6.72)	0.50 (6.57)	0.15 (7.37)	2.26 (6.51)	0.99	0.598	**1.04**	**0.010**	**0.96**	**0.015**
Psychological distress	15.28 (5.39)	13.94 (4.24)	17.94 (6.09)	18.20 (6.71)	**1.16**	**<0.001**	**1.16**	**<0.001**	0.99	0.745
Anxiety symptoms	2.99 (2.83)	2.34 (2.43)	4.17 (2.93)	4.50 (3.31)	**1.28**	**<0.001**	**1.32**	**<0.001**	0.97	0.431
Number of daily hassles	5.07 (3.93)	4.31 (3.57)	6.36 (3.84)	6.95 (4.46)	**1.15**	**<0.001**	**1.18**	**<0.001**	0.97	0.278
*Individual enabling factors*										
	% (SD)	% (SD)	% (SD)	% (SD)						
% change in HRQOL	10.19 (66.89)	13.57 (78.44)	0.89 (27.74)	5.55 (32.80)	**0.99**	**0.005**	1.00	0.227	0.99	0.147
% change in life satisfaction	1.78 (18.03)	2.30 (16.34)	2.92 (19.77)	-1.62 (22.31)	1.00	0.736	**0.99**	**0.028**	**1.02**	**0.037**
	Mean (SD)	Mean (SD)	Mean (SD)	Mean (SD)						
Costs – societal perspective ($CAN)	14,057 (15,223)	13,088 (13,404)	15,933 (16,990)	16,228 (19,727)	**1.01**	**0.049**	**1.01**	**0.036**	1.00	0.901
Social deprivation index	3.18 (1.39)	3.11 (1.40)	3.27 (1.39)	3.34 (1.32)	1.09	0.257	1.12	0.118	0.97	0.714
Material deprivation index	2.96 (1.35)	2.97 (1.35)	2.96 (1.34)	2.89 (1.38)	0.99	0.918	0.96	0.547	1.04	0.689
Social support	2.79 (0.57)	2.79 (0.59)	2.82 (0.48)	2.80 (0.57)	1.12	0.530	1.03	0.862	1.09	0.714
Continuity of care index	0.30 (0.24)	0.32 (0.25)	0.30 (0.24)	0.26 (0.21)	0.73	0.444	**0.32**	**0.015**	2.28	0.147
Treatment of CMD	n (%)	n (%)	n (%)	n (%)						
No CMD	533 (76)	397 (82)	60 (58)	76 (71.0)	REF		REF		REF	
Inadequate care	101 (15)	68 (14)	17 (17)	16 (15.0)	1.65	0.098	1.23	0.498	1.35	0.445
Adequate care	63 (9)	22 (4)	26 (25)	15 (14.0)	**7.82**	**<0.001**	**3.56**	**<0.001**	**2.20**	**0.032**
*Health system enabling factors*										
Type of primary care practices	n (%)	n (%)	n (%)	n (%)						
Small clinics (< 3 GP)	224 (29.6)	141 (28)	42 (33)	41 (34)	1.29	0.228	1.38	0.141	0.94	0.819
Large clinics (≥ 3 GP)	533 (70.4)	369 (72)	85 (67)	78 (66)	REF		REF		REF	
	Mean (SD)	Mean (SD)	Mean (SD)	Mean (SD)						
Attraction index – general services	48.39 (31.48)	47.32 (30.48)	51.89 (33.34)	49.31 (33.60)	1.00	0.141	1.00	0.525	1.00	0.531
Attraction index – psychiatric services	5.22 (8.15)	4.87 (7.57)	5.96 (9.38)	5.95 (9.05)	1.02	0.169	1.02	0.186	1.00	0.991
Retention index – general services	0.64 (0.19)	0.65 (0.20)	0.66 (0.19)	0.62 (0.19)	1.50	0.447	0.58	0.288	2.56	0.151
Retention index – psychiatric services	0.31 (0.35)	0.31 (0.35)	0.30 (0.35)	0.33 (0.35)	0.97	0.988	1.25	0.440	0.79	0.520
*Individual predisposing factors*										
Sex										
Female	434 (56)	272 (52)	88 (69)	74 (61)	**2.01**	**<0.001**	1.41	0.093	1.43	0.181
Male	337 (44)	249 (48)	40 (31)	48 (39)	REF		REF		REF	
	Mean (SD)	Mean (SD)	Mean (SD)	Mean (SD)						
Age	72.86 (5.64)	72.86 (5.51)	73.10 (5.76)	72.62 (6.10)	1.01	0.669	0.99	0.673	1.02	0.504
Marital status										
Married	507 (66)	348 (67)	79 (62)	80 (66)	REF		REF		REF	
Not married	264 (34)	173 (33)	49 (38)	42 (34)	1.25	0.279	1.06	0.797	1.18	0.527
Education										
0-7 years	132 (17)	99 (19)	17 (13)	16 (13)	REF		REF		REF	
+8 years	638 (83)	421 (81)	111 (87)	106 (87)	1.54	0.128	1.56	0.125	0.99	0.969
Presence of adverse childhood experience										
Yes	87 (11)	47 (9)	19 (15)	21 (17)	1.76	0.052	**2.10**	**0.009**	0.84	0.610
No	684 (89)	474 (91)	109 (85)	101 (83)	REF		REF		REF	

^a^ Sample sizes vary according to study variable. The largest sample size is presented. There was missing data for the following continuous variables: deprivation indexes (29), education (1), cognitive decline (2), continuity of care (1), attraction index for psychiatric services (14), change in HRQOL (1), change in satisfaction with life (8).

Significant results are in bold.

CI: Confidence intervals; CMD: common mental disorders; GP: general practitioners; HRQOL: Health-related quality of life; SD: standard deviation

**Table 3.** Factors associated with perceived need for mental health care in the overall sample (n=759)

	Met need vs no need (ref)		Unmet need vs no need (ref)		Met need vs unmet need (ref)	
	Adjusted odds ratio (95% CI)	p value	Adjusted odds ratio (95% CI)	p value	Adjusted odds ratio (95% CI)	p value
*Individual need factors*						
Course of CMD						
No disorder	REF		REF		REF	
Incident cases	**2.77 (1.30 to 5.94)**	**0.009**	1.03 (0.44 to 2.40)	0.942	**2.69 (1.07 to 6.77)**	**0.036**
Remitted cases	**1.81 (1.06 to 3.12)**	**0.030**	1.09 (0.61 to 1.92)	0.780	1.68 (0.85 to 3.30)	0.135
Persistent cases	**5.39 (2.47 to 11.76)**	**<0.001**	1.46 (0.56 to 3.84)	0.441	**3.68 (1.43 to 9.51)**	**0.007**
Number of chronic physical diseases						
<3 physical disorders	**REF**		REF		REF	
≥3 physical disorders	**1.90 (1.10 to 3.28)**	**0.022**	0.72 (0.44 to 1.18)	0.189	**2.63 (1.36 to 5.08)**	**0.004**
% cognitive decline	0.99 (0.95 to 1.02)	0.369	**1.04 (1.01 to 1.07)**	**0.023**	**0.95 (0.91 to 0.99)**	**0.011**
Psychological distress	1.06 (0.99 to 1.15)	0.099	1.07 (1.00 to 1.16)	0.060	0.99 (0.91 to 1.08)	0.834
Anxiety symptoms	1.07 (0.94 to 1.23)	0.321	1.11 (0.97 to 1.28)	0.125	0.96 (0.83 to 1.12)	0.622
Number of daily hassles	1.00 (0.94 to 1.07)	0.926	1.05 (0.98 to 1.13)	0.136	0.95 (0.88 to 1.03)	0.241
*Individual enabling factors*						
% change in HRQOL	**0.99 (0.98 to 0.99)**	**0.036**	1.00 (0.99 to 1.00)	0.372	0.99 (0.99 to 1.00)	0.235
% change in life satisfaction	1.00 (0.99 to 1.01)	0.574	0.99 (0.98 to 1.01)	0.288	1.01 (1.00 to 1.03)	0.169
Costs – societal perspective ($CAN)	1.00 (0.99 to 1.02)	0.953	1.00 (0.99 to 1.02)	0.599	1.00 (0.98 to 1.01)	0.691
Continuity of care index	0.66 (0.27 to 1.65)	0.378	**0.36 (0.14 to 0.97)**	**0.043**	1.84 (0.57 to 5.92)	0.309
*Individual predisposing factors*						
Sex						
Female	1.49 (0.95 to 2.35)	0.081	1.17 (0.75 to 1.82)	0.489	1.28 (0.73 to 2.24)	0.392
Male	REF		REF		REF	
Presence of adverse childhood experience						
Yes	1.45 (0.78 to 2.70)	0.245	1.72 (0.94 to 3.13)	0.078	0.84 (0.41 to 1.73)	0.643
No	REF		REF		REF	

Significant results are in bold.

CI: Confidence intervals; CMD: common mental disorders; GP: general practitioners; HRQOL: Health-related quality of life

**Table 4.** Factors associated with perceived need for mental health care in the subsample of older adults with a common mental disorder (n=160)

	Met need vs no need (ref)		Unmet need vs no need (ref)		Met need vs unmet need (ref)	
	Adjusted odds ratio (95% CI)	p value	Adjusted odds ratio (95% CI)	p value	Adjusted odds ratio (95% CI)	p value
*Individual need factors*						
Course of CMD						
Remitted cases	REF		REF		REF	
Persistent cases	**4.33 (1.64 to 11.36)**	**0.003**	1.96 (0.61 to 6.29)	0.259	2.21 (0.70 to 6.99)	0.179
Number of chronic physical diseases						
≥3 physical disorders	1.24 (0.41 to 3.79)	0.701	1.00 (0.24 to 4.22)	0.998	1.24 (0.28 to 5.46)	0.776
<3 physical disorders	REF		REF		REF	
% cognitive decline	0.97 (0.92 to 1.03)	0.289	1.01 (0.94 to 1.08)	0.868	0.96 (0.90 to 1.04)	0.326
Psychological distress	1.06 (0.91 to 1.24)	0.459	1.12 (0.95 to 1.33)	0.182	0.95 (0.80 to 1.12)	0.515
Anxiety symptoms	1.13 (0.85 to 1.48)	0.402	1.14 (0.83 to 1.56)	0.431	0.99 (0.72 to 1.36)	0.952
Number of daily hassles	0.95 (0.83 to 1.08)	0.411	1.09 (0.94 to 1.26)	0.264	0.87 (0.75 to 1.01)	0.061
*Individual enabling factors*						
% change in HRQOL	0.99 (0.98 to 1.01)	0.341	0.99 (0.97 to 1.01)	0.258	1.00 (0.98 to 1.03)	0.720
% change in life satisfaction	1.01 (0.99 to 1.04)	0.322	0.99 (0.96 to 1.02)	0.341	1.03 (1.00 to 1.06)	0.071
Costs – societal perspective ($CAN)	0.99 (0.96 to 1.02)	0.413	0.98 (0.94 to 1.01)	0.155	1.02 (0.98 to 1.05)	0.450
Adequacy of care						
Yes	**5.43 (2.09 to 14.29)**	**<0.001**	**3.73 (1.16 to 12.05)**	**0.027**	1.46 (0.44 to 4.81)	0.535
No	REF		REF		REF	
Continuity of care index	1.41 (0.21 to 9.50)	0.722	0.50 (0.05 to 4.81)	0.545	2.85 (0.28 to 29.51)	0.380
*Individual predisposing factors*						
Sex						
Female	0.96 (0.35 to 2.62)	0.937	0.71 (0.21 to 2.37)	0.573	1.36 (0.36 to 5.14)	0.650
Male	REF		REF		REF	
Presence of adverse childhood experience						
Yes	0.88 (0.23 to 3.46)	0.861	0.69 (0.12 to 4.00)	0.679	1.28 (0.22 to 7.41)	0.780
No	REF		REF		REF	

Significant results are in bold.

CI: Confidence intervals; CMD: common mental disorders; GP: general practitioners; HRQOL: Health-related quality of life

### Need versus no Need

The multivariable analyses (Table S3) showed that, as compared with those with no CMD, individuals with persistent CMD were three times more likely to report a need for care in the total and subsample, while those with a CMD and a need for care were more likely to receive adequate care (Adjusted odds ratio [AOR] 5.38, CI 2.02–14.29, *p* < 0.001).

### Met versus no Need

In the total sample, participants with a met need were more likely to report three or more physical disorders at follow-up (AOR 1.90, CI 1.10–3.28,   *p = *0.022) and a CMD at baseline and/or follow-up, and to report a decrease in their HRQOL (AOR 0.99, CI 0.98–0.99,   *p = *0.036). In the subsample of those with a CMD, individuals reporting a met need were more likely to have a persistent CMD (AOR 4.33, CI 1.64–11.36,   *p = *0.003) and receive adequate care (AOR 5.43, CI 2.09–14.29, *p* < 0.001).

### Unmet versus no Need

In the total sample, participants with an unmet need were more likely to report cognitive decline (AOR 1.04, CI 1.01–1.07,   *p = *0.023) and lower continuity of care (AOR 0.36, CI 0.14–0.97,   *p = *0.043). In those with a CMD at baseline, those categorized as receiving adequate care at baseline were more likely to also report an unmet need (AOR 3.73, CI 1.16–12.05,   *p = *0.027).

### Met versus Unmet Need

In the total sample, older adults with met need, although less likely to show cognitive decline (AOR 0.95, CI 0.91–0.99,   *p = *0.011), were more likely to report at least three physical disorders (AOR 2.63, CI 1.36–5.08,   *p = *0.004) and have an incident (AOR 2.69, CI 1.07–6.77,   *p = *0.036) and persistent CMD (AOR 3.68, CI 1.43–9.51,   *p = *0.007).

## Discussion

The findings of the current study show that one in three community-living primary care older adults and one in two with a CMD report a need for mental health care. In the overall sample, older adults reporting a met need, versus unmet and no need, were more likely to have a CMD at baseline and/or follow-up. Sherbourne et al.’s secondary analysis of data from a randomized control trial also showed that adults with depression receiving adequate treatment and reporting unmet needs for care were more likely to have a persistent disorder at 18 and 24 month follow-up.^8^ Older adults with met needs received care, which may have increased their likelihood to be diagnosed for their symptoms and be identified as persistent cases.

In individuals with a CMD at baseline, a need for care (met or unmet need) versus no need was associated with an increased likelihood of having had received in the past minimally adequate mental health care. Similarly, a primary care study in the Netherlands showed that adults with either depression or anxiety receiving guideline-concordant treatment were more likely to perceive a need for medication, counseling or referral to specialized care.^60^ The definition of minimally adequate treatment for CMD in the current study included outpatient medial consultations, pharmacological treatment and therapy consultations. Although adequacy of care and PNC were measured at different times, receiving minimally adequate care might have in part increased the likelihood in the future of participants receiving follow-up care meeting or not their perceived needs.

The findings of the current study also showed, in the overall sample, that those with a met need, as compared to others, were more likely to report three or more chronic diseases. This is consistent with previous studies,^3,61^ suggesting that physical conditions increase the likelihood of receiving care and therefore the chances of having a mental health care need met. Also of interest, individuals reporting an unmet need were more likely to report cognitive decline, whereas those reporting a met versus unmet need reported reduced cognitive decline. Although all participants were cognitively competent at baseline and follow-up, it is possible that participants referred to their cognitive impairment symptoms when referring to their mental health. A cross-sectional study in primary care older adults from the larger ESA-Services cohort showed that reduced cognitive functioning was associated with low satisfaction with continuity of care, patient–provider interactions and adequacy of care, and this while controlling for a number of clinical and socio-demographic factors.^62^ Additional bivariate analyses showed that continuity of care in the 3 years following baseline was not associated with cognitive decline at follow-up. The results may in part reflect the lack of primary care follow-up received by older adults reporting cognitive decline, which may have further influenced their cognitive impairment and results in reporting unmet mental health needs. Given the limited number of studies, future research is needed to better understand the association between cognitive impairment and functioning and perceived need for mental health care in older adults.

In the overall sample, individuals with met need as compared with no need were also more likely to report a decrease in HRQOL between baseline and follow-up. This was not observed for those with unmet need. To our knowledge, no study has looked at the association between PNC and change in quality of life.

The current study did not find an association between costs associated with health service use and perceived need for mental health care. Similar results have been previously reported among adults with depression or anxiety.^10^. From a societal perspective, including patient preferences and needs could help to meet patient mental health needs for care, while also optimising the allocation of mental health resources.

In summary, the current findings showed that older adults with unmet needs were more likely to report cognitive decline, have lower continuity of care and receive adequate care for CMD. From a clinical perspective, greater efforts need to be taken to better manage the care of older adults with cognitive impairment to meet their mental health needs and delay further cognitive decline. Further, additional analyses in those with a CMD showed that the need for therapy was the most needed type of help reported by 48% of older adults with unmet needs. Although the effectiveness of psychotherapy has been shown for the treatment of CMD,^49,63^ their accessibility in Quebec and Canada have been limited for individuals without private insurance.^64,65^ This highlights the increased need for a publicly funded universal program to improve access to psychotherapy. Participants reporting a met need were more likely to present with a more complex clinical profile including the presence of a CMD and multiple physical chronic disorders and lower HRQOL. These findings may in part be explained by the fact that up to 40% of adults with CMD and other co-morbidities do not adequately respond to treatment.^66,67^ Outcomes of interest for older adults with chronic depression or anxiety may be focused toward improved functioning and life fulfillment and satisfaction^67^. Finally, 54% of older adults with a CMD did not perceive a need for care despite having mental health symptoms, which calls for research focusing on factors including coping mechanisms, spontaneous remission and the use of other types of help for their symptoms (e.g. exercise) to better explain these results.

Overall, physicians need to focus on individuals with cognitive impairment and lower continuity of care, which was associated with unmet mental health needs. Improved follow-up in these populations may improve health care needs and outcomes.

The findings of the current study need to be considered with the following limitations. First, this cohort study was conducted among older adults who accepted the linking of their health survey to medico-administrative data. Additional analyses showed that participants who accepted and did not accept the linking of their data were similar in terms of PNC and socio-demographic factors, thus limiting the potential selection bias. Fifty percent of the study sample was lost to follow-up. These participants were older, less educated, had lower cognitive functioning as well as higher psychological distress and number of chronic diseases and anxiety symptoms. Given that losses had more complex clinical profiles, the associations between PNC and associated health-related outcomes might have been underestimated. A classification bias may also be present as physicians are not required to register diagnoses to be paid by the RAMQ, resulting in the potential under-reporting of mental health diagnoses in administrative database as previously suggested in a Canadian report.^69^ This could be even more problematic in older adults with multiple chronic diseases.^69^ To minimize this potential information bias, we combined self-reported and administrative data to assess the presence of CMD. Previous studies have reported low concordance between self-reported and diagnosed CMD and suggested using both data sources when available.^13, 70^ Depression and anxiety disorders and PNC were measured in the last 6 and 12 months during a face-to-face interview, suggesting the presence of potential desirability and memory bias. Further, societal costs included the portion of medication costs covered under the provincial public drug plan and paid by beneficiaries. A total of 100 participants were not covered under Quebec’s drug plan or were not covered for the entire study period. Out-of-pocket costs paid for private insurance were not considered in this study. No difference was observed between participants covered and not covered according to the presence of PNC, and more specifically, perceived need for medication, suggesting that the potential information bias is non-differential. Medical visits in the RAMQ database are based on physician billing and it is not always possible to differentiate whether the visit was a follow-up or assessment. However, the results seem to show high continuity of care reflecting the potential for physicians to be able to assess changes in health status. Finally, the results of the current study are only generalizable to older adults consulting in primary care clinics and living at home without significant cognitive impairment.

## Authors’ Note

The authors are not legally authorised to share or publicly publish linked survey and health administrative data owing to privacy or ethical restrictions related to the use of administrative provincial health data. Participants were not requested to give informed consent for data sharing.

The province of Québec’s “Commission d’Accès à l’Information” gave approval to merge these datasets. Requests for access to the data should be addressed to the research ethics committee of the CIUSSS de l’Estrie-CHUS.

## Supplemental Material

sj-docx-1-cpa-10.1177_07067437211055430 - Supplemental material for Perceived Need for Mental Health Care and Associated Factors and Outcomes in Older Adults Consulting in Primary Care Click here for additional data file. Supplemental material, sj-docx-1-cpa-10.1177_07067437211055430 for Perceived Need for Mental Health Care and Associated Factors and Outcomes in Older Adults Consulting in Primary Care by Catherine Lamoureux-Lamarche, Djamal Berbiche and Helen-Maria Vasiliadis in The Canadian Journal of Psychiatry

## References

[1] Institute of Medicine Committee on Quality of Health Care. Crossing the quality chasm: a new health system for the 21st century. In: Crossing the quality chasm: A new health system for the 21st century. Washington, DC: National Academies Press; 2001.

[2] Prins MA . Mental health care from the patient's perspective: A study of patients with anxiety and depression in general practice. NIVEL; 2010.

[3] Sunderland A , Findlay LC. Perceived need for mental health care in Canada: Results from the 2012 Canadian community health survey—mental health. Statistics Canada, Ottawa; 2013. 24258361

[4] Seekles W Cuijpers P Van de Ven P , Penninx BW, Verhaak PF, Beekman AT, van Straten A. Personality and perceived need for mental health care among primary care patients. J Affect Disord. 2012;136(3):666-74. 22104392 10.1016/j.jad.2011.10.009

[5] Mackenzie CS Pagura J Sareen J . Correlates of perceived need for and use of mental health services by older adults in the collaborative psychiatric epidemiology surveys. Am J Geriatr Psychiatry. 2010;18(12):1103-15. 20808105 10.1097/JGP.0b013e3181dd1c06 PMC2992082

[6] Fleury MJ Grenier G Bamvita JM , Perreault M, Caron J. Variables associated with perceived unmet need for mental health care in a Canadian epidemiologic catchment area. Psychiatr Serv. 2016;67(1):78-85. 26325455 10.1176/appi.ps.201400363

[7] Dezetter A Duhoux A Menear M , Roberge P, Chartrand E, Fournier L. Reasons and determinants for perceiving unmet needs for mental health in primary care in Quebec. Can J Psychiatry. 2015;60(6):284-93. 26175326 10.1177/070674371506000607 PMC4501586

[8] Sherbourne C Schoenbaum M Wells KB , Croghan TW. Characteristics, treatment patterns, and outcomes of persistent depression despite treatment in primary care. Gen Hosp Psychiatry. 2004;26(2):106-14. 15038927 10.1016/j.genhosppsych.2003.08.009

[9] Sareen J Stein MB Campbell DW , Hassard T, Menec V. The relation between perceived need for mental health treatment, DSM diagnosis, and quality of life: A Canadian population-based survey. Can J Psychiatry. 2005;50(2):87-94. 15807224 10.1177/070674370505000203

[10] Prins M Bosmans J Verhaak P , van der Meer K, van Tulder M, van Marwijk H, Laurant M, Smolders M, Penninx B, Bensing J. The costs of guideline-concordant care and of care according to patients’ needs in anxiety and depression. J Eval Clin Pract. 2011;17(4):537-46. 20586845 10.1111/j.1365-2753.2010.01490.x

[11] Andersen RM . Revisiting the behavioral model and access to medical care: Does it matter? J Health Social Behav. 1995;36(1):1-10. 7738325

[12] Barnett K Mercer SW Norbury M , Watt G, Wyke S, Guthrie B. Epidemiology of multimorbidity and implications for health care, research, and medical education: A cross-sectional study. Lancet. 2012;380 (9836):37-43. 10.1016/S0140-6736(12)60240-2 22579043

[13] Guerra S Gontijo Berbiche D Vasiliadis HM . Measuring multimorbidity in older adults: Comparing different data sources. BMC Geriatr. 2019; 19(1): 166. 31200651 10.1186/s12877-019-1173-4 PMC6570867

[14] Preville M Vasiliadis HM Boyer R , Goldfarb M, Demers K, Brassard J, Béland SG; Scientific Committee of the ESA Study. Use of health services for psychological distress symptoms among community-dwelling older adults. Can J Aging. 2009; 28(1):51-61. 19860966 10.1017/S0714980809090011

[15] Canada Statistics . Population projections for Canada, provinces and territories 2009 to 2036. Ontario, Canada: Statistics Canada; 2010.

[16] Préville M Mechakra-Tahiri SD Vasiliadis H-M , Mathieu V, Quesnel L, Gontijo-Guerra S, Lamoureux-Lamarche C, Berbiche D. Family violence among older adult patients consulting in primary care clinics: Results from the ESA (Enquête sur la santé des aînés) services study on mental health and aging. Can J Psychiatry. 2014;59(8):426-33. 25161067 10.1177/070674371405900805 PMC4143299

[17] Milan R Vasiliadis H-M . The association between side effects and adherence to antidepressants among primary care community-dwelling older adults. Aging Mental Health. 2020; 24(8): 1229-1236 10.1080/13607863.2019.1594165 30938182

[18] Preville M Boyer R Grenier S , Dubé M, Voyer P, Punti R, Baril MC, Streiner DL, Cairney J, Brassard J; Scientific Committee of the ESA Study. The epidemiology of psychiatric disorders in Quebec's older adult population. Can J Psychiatry. 2008;53(12):822-32. 19087480 10.1177/070674370805301208

[19] Folstein MF Folstein SE McHugh PR . “Mini-mental state”: A practical method for grading the cognitive state of patients for the clinician. J Psychiatr Res. 1975;12(3):189-98. 1202204 10.1016/0022-3956(75)90026-6

[20] Hudon C Potvin O Turcotte M-C , D'Anjou C, Dubé M, Préville M, Brassard J. Normalisation du mini-mental state examination (MMSE) chez les Québécois francophones âgés de 65 ans et plus et résidant dans la communauté. Can J Aging. 2009;28(4):347-57. 19925700 10.1017/S0714980809990171

[21] Meadows G Harvey C Fossey E , Burgess P. Assessing perceived need for mental health care in a community survey: Development of the perceived need for care questionnaire (PNCQ). Soc Psychiatry Psychiatr Epidemiol. 2000;35(9):427-35. 11089671 10.1007/s001270050260

[22] Canada Statistics . Enquête sur la santé dans les collectivités canadiennes (ESCC)—Santé mentale—questionnaire; 2013. Available from: http://www23.statcan.gc.ca/imdb-bmdi/instrument/5105_Q1_V3-fra.pdf

[23] American Psychiatric Association. Diagnostic and statistical manual of mental disorders (DSM-5^®^). Arlington, VA: American Psychiatric Publishing; 2013.

[24] Sewitch MJ Blais R Rahme E , Galarneau S, Bexton B. Pharmacologic response to a diagnosis of late-life depression: A population study in Quebec. Can J Psychiatry. 2006;51(6):363-70. 16786817 10.1177/070674370605100605

[25] Alaghehbandan R MacDonald D Barrett B , Collins K, Chen Y. Using administrative databases in the surveillance of depressive disorders—case definitions. Pop Health Manage. 2012;15(6):372-80. 10.1089/pop.2011.0084 22788998

[26] Régie de l’Assurance Maladie du Québec. Répertoire des diagnostics—CIM-9; 2020. Available from: https://www.ramq.gouv.qc.ca/fr/professionnels/medecins-specialistes/facturation/repertoire-diagnostics/Pages/cim-9_par-code.aspx

[27] Régie de l’Assurance Maladie du Québec. Répertoire des diagnostics—CIM-10; 2020. Available from: https://www.ramq.gouv.qc.ca/fr/professionnels/medecins-specialistes/facturation/repertoire-diagnostics/Pages/cim-10_par-code.aspx.

[28] Kessler RC Andrews G Colpe LJ , Hiripi E, Mroczek DK, Normand SL, Walters EE, Zaslavsky AM. Short screening scales to monitor population prevalences and trends in non-specific psychological distress. Psychol Med. 2002;32(6):959-76. 12214795 10.1017/s0033291702006074

[29] Spitzer RL Kroenke K Williams JB , Löwe B. A brief measure for assessing generalized anxiety disorder: The GAD-7. Arch Intern Med. 2006;166(10):1092-97. 16717171 10.1001/archinte.166.10.1092

[30] Vézina J , Giroux L. L’Échelle des Embêtements: Une étude de validation et d’adaptation du Hassles Scale pour une population adulte âgée. Paper presented at Meeting of the Canadian Psychological Association, Montreal, June 1988.

[31] Hickey A, Barker M, McGee H, O'Boyle C. Measuring health-related quality of life in older patient populations: a review of current approaches. Pharmacoeconomics. 2005;23(10):971-93. 10.2165/00019053-200523100-00002 16235972

[32] Diener E Emmons RA Larsen RJ , Griffin S. The satisfaction with life scale. J Pers Assess. 1985;49(1):71-5. 16367493 10.1207/s15327752jpa4901_13

[33] Blais MR Vallerand RJ Pelletier LG , Brière NM. L'échelle de satisfaction de vie: Validation canadienne-française du “Satisfaction with Life Scale”. Can J Behav Sci/Rev Can Sci Comport. 1989; 21(2): 210.

[34] Vasiliadis H-M Latimer E Dionne P-A , Préville M. The costs associated with antidepressant use in depression and anxiety in community-living older adults. Can J Psychiatry. 2013;58(4):201-9. 23547643 10.1177/070674371305800405

[35] Vasiliadis H-M Dionne PA Preville M , Gentil L, Berbiche D, Latimer E. The excess healthcare costs associated with depression and anxiety in elderly living in the community. Am J Geriat Psychiatry. 2013;21(6):536-48. 10.1016/j.jagp.2012.12.016 23567409

[36] Canadian Agency for Drugs and Technologies for Health (CADTH). Guidelines for the economic evaluation of health technologies: Canada. 4th ed. Ottawa, Ontario: CADTH; 2017.

[37] Canadian Agency for Drugs Technologies in Health. Guidelines for the economic evaluation of health technologies: Canada. In: Guidelines for the economic evaluation of health technologies: Canada. CADTH; 2006.

[38] Reinharz D Lesage AD Contandriopoulos AP . Cost-effectiveness analysis of psychiatric deinstitutionalization. Can J Psychiatry. 2000; 45(6): 533-8. 10986570 10.1177/070674370004500603

[39] Drummond MF Sculpher MJ Claxton K , Stoddart GL, Torrance, GW. Methods for the economic evaluation of health care programmes. Oxford: Oxford University Press; 2015.

[40] Ministère de la Santé et des Services Sociaux (MSSS). AS-471—Rapports financiers annuels des établissements. MSSS; 2018. Available from: https://www.donneesquebec.ca/recherche/fr/dataset/as-471-rapports-financiers-annuels-des-etablissements/resource/ffc05092-520b-4383-ab87-292443a8cbd0.

[41] Ministère de la Santé et des Services Sociaux (MSSS). Liste par centre d’activités MSSS. Available from: http://msssa4.msss.gouv.qc.ca/fr/document/d26ngest.nsf/lca?OpenView.

[42] Rosenheck R Frisman L Neale M . Estimating the capital component of mental health care costs in the public sector. Admin Policy Mental Health Mental Health Serv Res. 1994; 21(6): 493-509.

[43] Canada Government of . Classes of depreciable property. Government of Canada; 2020. Available from: https://www.canada.ca/en/revenue-agency/services/tax/businesses/topics/sole-proprietorships-partnerships/report-business-income-expenses/claiming-capital-cost-allowance/classes-depreciable-property.html.

[44] Le Commissaire de la Santé et au Bien-Être. Les urgences au Québec: Évolution de 2003–2004 à 2012-2013 Governement du Québec; 2014.

[45] Commission des normes dle, de la sante et de la securité du travail. History of the minimum wage; 2020. Available from: https://www.cnt.gouv.qc.ca/en/wages-pay-and-work/wages/history-of-the-minimum-wage/index.html.

[46] Gamache P Hamel D , Pampalon R. L’indice de défavorisation matérielle et sociale: en bref. INSPQ; 2015.

[47] Canada Statistics . Canadian community health survey (CCHS)—2012; 2014. Available from: https://www23.statcan.gc.ca/imdb-bmdi/instrument/3226_Q1_V9-eng.htm.

[48] Bice TW Boxerman SB . A quantitative measure of continuity of care. Med Care; 1977; 15(4): 347-9. 859364 10.1097/00005650-197704000-00010

[49] Katzman MA Bleau P Blier P , Chokka P, Kjernisted K, Van Ameringen M & the Canadian Anxiety Guidelines Initiative Group on behalf of the Anxiety Disorders Association of Canada. Canadian clinical practice guidelines for the management of anxiety, posttraumatic stress and obsessive–compulsive disorders. BMC Psychiatry. 2014;14(1):S1. 25081580 10.1186/1471-244X-14-S1-S1 PMC4120194

[50] MacQueen GM Frey BN Ismail Z , Jaworska N, Steiner M, Lieshout RJ, Kennedy SH, Lam RW, Milev RV, Parikh SV, Ravindran AV; CANMAT Depression Work Group. Canadian Network for Mood and Anxiety Treatments (CANMAT) 2016 clinical guidelines for the management of adults with major depressive disorder: Section 6. Special populations: Youth, women, and the elderly. Can J Psychiatry. 2016;61(9):588-603. 27486149 10.1177/0706743716659276 PMC4994788

[51] Parikh SV Quilty LC Ravitz P , Rosenbluth M, Pavlova B, Grigoriadis S, Velyvis V, Kennedy SH, Lam RW, MacQueen GM, Milev RV, Ravindran AV, Uher R; CANMAT Depression Work Group. Canadian Network for Mood and Anxiety Treatments (CANMAT) 2016 clinical guidelines for the management of adults with major depressive disorder: Section 2. Psychological treatments. Can J Psychiatry 2016;61(9):524-39. 27486150 10.1177/0706743716659418 PMC4994791

[52] Wolitzky-Taylor KB Horowitz JD Powers MB , Telch MJ. Psychological approaches in the treatment of specific phobias: a meta-analysis. Clin Psychol Rev 2008;28(6):1021-37. 18410984 10.1016/j.cpr.2008.02.007

[53] Roberge P Fournier L Duhoux A , Nguyen CT, Smolders M. Mental health service use and treatment adequacy for anxiety disorders in Canada. Soc Psychiatry Psychiatr Epidemiol 2011;46(4):321-30. 20217041 10.1007/s00127-010-0186-2

[54] Turgeon M , Guénette L. Portrait de l'usage des antidépresseurs chez les adultes assurés par le régime public d'assurance médicaments du Québec: Rapport final. Conseil du médicament; 2011.

[55] Canadian Coalition for Senior’s Mental Health. National Guidelines for Seniors’ Mental Health. Toronto, ON: Canadian Coalition for Seniors’ Mental Health 2006.

[56] Duhoux A Fournier L Gauvin L , Roberge P. Quality of care for major depression and its determinants: A multilevel analysis. BMC Psychiatry. 2012;12:142. 10.1186/1471-244X-12-142 PMC3544698 22985262

[57] Mireault J Lemay A . Analyses cliniques des hospitalisations de la population de ville de Laval et des patients de la Cité de la Santé de Laval, Montréal. Association des hôpitaux du Québec; 1999;113.

[58] Canada Statistics . Canadian Community Health Survey (CCHS)—Mental Health; 2012. Available from: http://www23.statcan.gc.ca/imdb-bmdi/instrument/5105_Q1_V3-eng.pdf.

[59] Van Buuren S Van Rijckevorsel JL . Imputation of missing categorical data by maximizing internal consistency. Psychometrika. 1992;57(4):567-80.

[60] Prins MA Verhaak PF Smolders M , Laurant MG, van der Meer K, Spreeuwenberg P, van Marwijk HW, Penninx BW, Bensing JM. Patient factors associated with guideline-concordant treatment of anxiety and depression in primary care. J Gen Intern Med. 2010; 25(7): 648-55. 20049547 10.1007/s11606-009-1216-1 PMC2881973

[61] Sareen J Cox BJ Afifi TO , Clara I, Yu BN. Perceived need for mental health treatment in a nationally representative Canadian sample. Can J Psychiatry. 2005; 50(10): 643-51. 16276856 10.1177/070674370505001011

[62] Pitrou I Berbiche D Vasiliadis HM . Mental health and satisfaction with primary care services in older adults: A study from the patient perspective on four dimensions of care. Fam Pract. 2020; 37(4): 459-464. 10.1093/fampra/cmaa019 32201895

[63] Canadian Coalition for Senior’s Mental Health & Canadian Academy of Geriatric Psychiatry. 2021 guideline update—Canadian guidelines on prevention, assessment and treatment of depression among older adults. Markham, Ontario: Canadian Coalition for Seniors’ Mental Health; 2021.

[64] Institut National de Santé et des Services Sociaux. Accès équitable aux services de psychothérapie au Québec. Québec: INESSS; 2017.

[65] Vasiliadis H-M Dezetter A . Publicly funded programs of psychotherapy in Australia and England. Sante Ment Que. 2015;40(4):101-18. 27203535

[66] Flint AJ . Treatment-resistant depression in late life. CNS Spectrums. 2002;7(10):733-8. 15034498 10.1017/s1092852900008725

[67] Van Dijk M Verbraak M Oosterbaan D , Hoogendoorn AW, van Balkom AJLM. Predictors of non-response and persistent functional impairments in treatment adhering to evidence-based practice guidelines for anxiety disorders. J Depression Anxiety. 2014;3(3):159.

[68] Gouvernement du Québec. (2015). Faire ensemble et autrement: Plan d'action en santé mentale 2015-2020. Québec (QC): Ministére de la Santé et des Services Sociaux.

[69] Public Health Agency of Canada. Report from the Canadian Chronic Disease Surveillance System: Mental illness in Canada 2015. Ottawa: Ontario Public Health Agency of Canada; 2015.

[70] Edwards J Thind A Stranges S , Chiu M, Anderson KK. Concordance between health administrative data and survey-derived diagnoses for mood and anxiety disorders. Acta Psychiatr Scand. 2020; 141(4): 385-95. 31883386 10.1111/acps.13143

