# Supplementary material for: Perceived Need for Mental Health Care and Associated Factors and Outcomes in Older Adults Consulting in Primary Care
Source: Can J Psychiatry. 2021 Nov 26;67(7):553–64. doi: 10.1177/07067437211055430 (PMC9234897; doi:10.1177/07067437211055430)
Supplement: sj-docx-1-cpa-10.1177_07067437211055430 - Supplemental material for Perceived Need for Mental Health Care and Associated Factors and Outcomes in Older Adults Consulting in Primary Care [file sj-docx-1-cpa-10.1177_07067437211055430.docx]

| **Table S1: Additional information on the measure of perceived need for mental health care** | |
| --- | --- |
| Type of help received | 1. In the last 12 months, did you receive the following types of help because of problems related to your emotions, mental health or alcohol consumption? 2. Information on these problems, their treatments or available services (yes/no) 3. Medications (yes/no) 4. Consultations, therapy or help regarding interpersonal relations (yes/no) 5. Others (specify) |
| Perceived adequacy of help received | 1. Do you think you received as much help as you needed (in the past 12 months)? 2. Yes 3. No |
| Perceived need for care | 1. Which of these types of help would you have needed more of (in the past 12 months)? 2. Information on these problems, their treatments or available services (yes/no) 3. Medications (yes/no) 4. Consultations, therapy or help regarding interpersonal relations (yes/no) 5. Others (specify) |

| **Table S2 : Description of study measures** | | |
| --- | --- | --- |
|  | Time | Description |
| *Individual need factors* | | |
| Course of CMD | Baseline, follow-up | No CMD: absence at baseline and follow-up  Remitted case: presence at baseline and absence at follow-up  Incident case: absence at baseline and presence at follow-up  Persistent case: presence at baseline and follow-up  Self-reported CMD included: major depression, social phobia, specific phobia, panic disorder, agoraphobia and generalized anxiety disorder^1^.  The presence of a physician diagnosis of CMD, in the 6 months  surrounding the baseline and follow-up, was assessed with the following International Classification of Diseases (ICD) 9^th^ and 10^th^ revisions codes: 300.0, 300.2, 311.0, 311.9, 300.4, F32, F33, F34.1, F40, F41^2-5^. |
| Number of chronic physical diseases | Follow-up | The number of chronic physical diseases was categorized as follow:  ≥3 physical disorders, <3 physical disorders. The number of chronic diseases was based on a list of 17 chronic diseases (high blood pressure, arthritis, heart disease, eye disease, back problems, gastrointestinal disease, thyroid disease, metabolism disorder, diabetes, anemia, hypercholesterolemia, respiratory disease, liver disease, kidney or urinary disease, skin disease, frequent headaches, cancer). This list has previously been used in epidemiological surveys conducted in older adults^6,7^. |
| % cognitive decline | Baseline, follow-up | Cognitive functioning was measured with the French-Canadian version of the Mini-Mental State Examination adapted for at home interviews^8,9^  The percentage of cognitive decline was calculated:  *((Score at baseline - Score at follow-up) / Score at baseline) * 100* |
| Psychological distress | Follow-up | Kessler Psychological Distress Scale (K10) ranging from 10-50, higher score indicating more severe symptoms^10^ |
| Anxiety symptoms | Follow-up | 7-item Generalized Anxiety Disorder (GAD-7) ranging from 0-21, higher score indicating more severe symptoms^11^ |
| Number of daily hassles | Follow-up | The number of daily hassles (0-22) was assessed based on a reduced list of 22 daily hassles measured with an adapted French version of the Hassles Scale^12^ |
| *Individual enabling factors* | | |
| % change in HRQOL | Baseline,  follow-up, | Vertical visual analog scale ranging from 0 to 100, representing the worst and best health state imaginable  The percentage of change was calculated:  *((Score at follow-up – Score at baseline)/Score at baseline) * 100*  The use of visual analog scales have been previously reported in samples of adults with CMD^13,14^ as well as in older populations^15,16^. |
| % change in life satisfaction | Baseline,  follow-up | 5-question adapted French version of the Satisfaction With Life Scale ranging from ‘’completely agree’’ to “completely disagree’’ with a total score varying from 5 to 25^17,18^  The percentage of change was calculated:  *((Score at follow-up – Score at baseline)/Score at baseline) * 100*  The original scale had good validity and reliability in older adults^18^. |
| Costs – societal perspective ($CAN) | 3 years after baseline | Total costs from the societal perspective were calculated in the 3 years following baseline and were based on guidelines on economic evaluation, published methodologies and the literature^19-23^. Costs from the health system perspective included those related to emergency department and outpatient visits, inpatient stays, medication costs covered by the public drug plan and physician fees. Costs measured from the patient perspective included fees paid out for drugs dispensed, fees paid for psychotherapy sessions in private practices and loss of productivity related to medical visits and inpatient stay. Health care utilization was assessed from the RAMQ and Med-ECHO databases. Unit costs were assessed based on 2013-2014 published financial reports from the Quebec Ministry of health and social services^24,25^ and data from the literature^19,26-30^. |
| Social deprivation index | Baseline | The area-level social and material deprivation indexes were calculated using the postal code of participants and were based on the following six factors : 1) the proportion of individuals aged 15 years and over who are not married or living with a partner, 2) who do not have a secondary diploma or certificat, 3) who are employed, 4) who are living alone, 5) the mean income of individuals aged 15 years and over and 6) the proportion of one-parent families^31^. The deprivation indexes varied from 1 (most deprivated) to 5 (most privated)^31^. |
| Material deprivation index |  |  |
| Social support | Follow-up | The presence of social support was measured by receiving at least one of the following three types of support: 1) help to resolve issues; 2) being able to discuss and confide to someone; et 3) help to fight discouragement and give affection^32^. |
| Continuity of care index | 3 years after baseline | 3 years continuity of ambulatory care based  on Bice-Boxerman index^33^ |
| Minimally adequate treatment | Baseline | Minimally adequate treatment was assessed at baseline from an epidemiological perspective^34^ using Canadian clinical guidelines and relevant literature^34-41^. Treatment adequacy for depression was defined by the presence of an antidepressant prescription and 4 medical consultations with a general practitioner (GP) within 3 months or 8 therapy or psychotherapy sessions in the 12 months following the diagnosis or first prescription or therapy session^36,37,40,41^. Adequacy of treatment for anxiety disorders was defined by the presence of a prescription of an antidepressant, anxiolytic or another recommended medication and 4 medical consultations with a GP within 3 months or 7 therapy or psychotherapy consultations in the 12 months following the diagnosis or first prescription or therapy session^35,39,42^. This definition was employed for all anxiety disorder understudied except for specific phobia. Given that pharmacological treatments are not recommended for this anxiety disorder, treatment adequacy was based on the presence of 5 therapy or psychotherapy consultations in the 12 months following the diagnosis or first prescription or therapy session^35,38^. Older adults receiving adequate care for depression or an anxiety disorder were classified as receiving adequate care. |
| *Health system enabling factors* | | |
| Type of primary care practices | Baseline | Type of primary care clinics where the participants were recruited  Small clinics (small private clinics with ≤3 physicians) vs  Large clinics (family medicine groups, local community health centers, private clinics with >3 physicians)  Family medicine groups and local community services centers (included in large clinics) are multidisciplinary practice settings that also have the opportunity to work closely with other health professionals including mental health resources (e.g. social workers, psychologists). Therefore, accessibility to mental health care could have been better in these types of clinics. |
| Attraction index – general services | Historical measures (2006) | Numerator: Adjusted costs of general or psychiatric services (inpatient and outpatient) based on physicians’ billing by the local population in a region^43^  Denominator: Total adjusted costs of general or psychiatric services (inpatient and outpatient) based on physicians’ billing by the local population despite the region the service was received^43^ |
| Attraction index – psychiatric services |  |  |
| Retention index – general services |  | Numerator: Adjusted costs of general or psychiatric services (inpatient and outpatient) based on physicians’ billing by the local population in a region^43^  Denominator: Total adjusted costs of general or psychiatric services (inpatient and outpatient) based on physicians’ billing rendered in this region regardless of the region of residence^43^ |
| Retention index – psychiatric services |  |  |
| *Individual predisposing factors* | | |
| Age | Baseline | Continuous variable |
| Sex | Baseline | Female vs male |
| Marital status | Baseline | Married/living with a partner vs not married/not living with a partner |
| Education | Baseline | Primary vs Secondary/post-secondary/university |
| Presence of adverse childhood experience | Follow-up | The presence of adverse childhood experience was assessed based on a list of 7 adverse events experienced before the age of 16 years old. The frequency of each adverse events being experienced ranged from  « never » to «more than 10 times »^44^. Participants who experienced at least one event at least one time where categorized has having an adverse childhood experience. |

| **Table S3: Bivariate and multivariable associations between the presence of a perceived need for care (met or unmet need) and study variables** | | | | | | |
| --- | --- | --- | --- | --- | --- | --- |
|  | **Bivariate analyses**  **Total sample**  **N=771** | | **Multivariable analyses**  **Total sample**  **N=704** | | **Multivariable analyses**  **Subsample**  **N=149** | |
|  | **Need vs no need (ref)** | | **Need vs**  **no need (ref)** | | **Need vs**  **no need (ref)** | |
|  | **Odds ratio** | **P-value** | **Odds ratio** | **P-value** | **Odds ratio** | **P-value** |
| *Individual need factors* | | | | | | |
| Course of CMD  No CMD  Incident cases  Remission cases  Persistent cases | REF  **3.41 (1.91-6.08)**  **1.85 (1.25-2.75)**  **6.23 (3.27-11.85)** | **<0.001**  **0.002**  **<0.001** | REF  1.90 (0.96-3.77)  1.34 (0.84-2.14)  **3.11 (1.51-6.39)** | 0.065  0.214  **0.002** | REF  **3.08 (1.23-7.69)** | **0.016** |
| Number of chronic physical diseases  <3 physical disorders  ≥3 physical disorders | REF  **1.74 (1.24-2.45)** | **0.001** | REF  1.20 (0.80-1.81) | 0.385 | REF  1.77 (0.56-5.52) | 0.328 |
| % cognitive decline | 1.02 (0.99-1.04) | 0.190 |  |  |  |  |
| Psychological distress | **1.16 (1.12-1.20)** | **<0.001** | 1.06 (1.00-1.13) | 0.056 | 1.12 (0.96-1.30) | 0.150 |
| Anxiety symptoms | **1.30 (1.22-1.38)** | **<0.001** | 1.12 (0.99-1.26) | 0.067 | 1.11 (0.85-1.45) | 0.438 |
| Number of daily hassles | **1.17 (1.12-1.21)** | **<0.001** | 1.02 (0.97-1.08) | 0.435 | 0.95 (0.84-1.09) | 0.485 |
| *Individual enabling factors* | | | | | | |
| % change in HRQOL | **0.99 (0.99-1.00)** | **0.011** | 1.00 (0.99-1.00) | 0.121 | 0.99 (0.98-1.01) | 0.370 |
| % change in life satisfaction | 1.00 (0.99-1.00) | 0.250 | 1.00 (0.99-1.01) | 0.865 | 1.00 (0.98-1.03) | 0.802 |
| Costs – societal perspective ($CAN) | **1.01 (1.00-1,02)** | **0.014** | 1.00 (0.99-1.01) | 0.740 | 0.98 (0.96-1.01) | 0.128 |
| Social deprivation index | 1.10 (0.99-1.24) | 0.081 | 1.02 (0.89-1.16) | 0.821 | 0.78 (0.57-1.08) | 0.133 |
| Material deprivation index | 0.97 (0.87-1.09) | 0.652 |  |  |  |  |
| Social support | 1.08 (0.82-1.41) | 0.599 |  |  |  |  |
| Continuity of care index | **0.50 (0.26-0.96)** | **0.038** | 0.48 (0.22-1.06) | 0.071 | 0.73 (0.11-5.06) | 0.753 |
| Treatment of CMD  No CMD  Inadequate care  Adequate care | REF  1.42 (0.90-2.24)  **5.44 (3.13-9.46)** | 0.137  **<0.001** |  |  | REF  **5.38 (2.02-14.29)** | **<0.001** |
| *Health system enabling factors* | | | | | | |
| Type of primary care practices  Small clinics (≤ 3 GP)  Large clinics (> 3 GP) | 1.33 (0.96-1.85)  REF | 0.086 | 1.27 (0.86-1.87)  REF | 0.225 | 0.74 (0.31-1.73)  REF | 0.484 |
| Attraction index – general services | 1.00 (1.00-1.01) | 0.172 |  |  |  |  |
| Attraction index – psychiatric services | 1.02 (1.00-1.03) | 0.087 | 1.01 (0.99-1.03) | 0.227 | 1.04 (0.98-1.09) | 0.201 |
| Retention index – general services | 0.93 (0.43-2.03) | 0.857 |  |  |  |  |
| Retention index – psychiatric services | 1.11 (0.72-1.70) | 0.642 |  |  |  |  |
| *Individual predisposing factors* | | | | | | |
| Sex  Female  Male | **1.69 (1.23-2.30)**  REF | **0.001** | 1.26 (0.87-1.82)  REF | 0.221 | 0.78 (0.30-2.02)  REF | 0.608 |
| Age | 1.00 (0.97-1.03) | 0.990 |  |  |  |  |
| Marital status  Married  Not married | REF  1.15 (0.84-1.58) | 0.382 |  |  |  |  |
| Education  0-7 years  +8 years | REF  **1.55 (1.01-2.38)** | **0.044** | REF  1.58 (0.97-2.58) | 0.069 | REF  2.65 (0.77-9.17) | 0.124 |
| Presence of adverse childhood experience  Yes  No | **1.92 (1.22-3.02)**  REF | **0.005** | 1.62 (0.98-2.70)  REF | 0.061 | 0.64 (0.17-2.40)  REF | 0.513 |
| Significant results are in bold.  CI: Confidence intervals; CMD: common mental disorders; GP: general practitioners; HRQOL: Health-related quality of life | | | | | | |

**References**

1. American Psychiatric Association. Diagnostic and statistical manual of mental disorders (DSM-5®). American Psychiatric Pub; 2013.

2. Sewitch MJ, Blais R, Rahme E, et al. Pharmacologic response to a diagnosis of late-life depression: A population study in Quebec. The Canadian Journal of Psychiatry 2006;51(6):363-370.

3. Alaghehbandan R, MacDonald D, Barrett B, et al. Using administrative databases in the surveillance of depressive disorders—case definitions. Population health management 2012;15(6):372-380.

4. Régie de l’Assurance Maladie du Québec. Répertoire des diagnostics - CIM-9. <https://www.ramq.gouv.qc.ca/fr/professionnels/medecins-specialistes/facturation/repertoire-diagnostics/Pages/cim-9_par-code.aspx>. Published 2020. Accessed 23 April, 2020.

5. Régie de l’Assurance Maladie du Québec. Répertoire des diagnostics - CIM-10. <https://www.ramq.gouv.qc.ca/fr/professionnels/medecins-specialistes/facturation/repertoire-diagnostics/Pages/cim-10_par-code.aspx>. Published 2020. Accessed 23 April, 2020.

6. Preville M, Boyer R, Grenier S, et al. The epidemiology of psychiatric disorders in Quebec's older adult population. Can J Psychiatry 2008;53(12):822-832.

7. Préville M, Mechakra-Tahiri SD, Vasiliadis H-M, et al. Family violence among older adult patients consulting in primary care clinics: results from the ESA (Enquête sur la santé des aînés) Services Study on Mental Health and Aging. The Canadian Journal of Psychiatry 2014;59(8):426-433.

8. Folstein MF, Folstein SE, McHugh PR. “Mini-mental state”: a practical method for grading the cognitive state of patients for the clinician. Journal of psychiatric research 1975;12(3):189-198.

9. Hudon C, Potvin O, Turcotte M-C, et al. Normalisation du Mini-Mental State Examination (MMSE) chez les Québécois francophones âgés de 65 ans et plus et résidant dans la communauté. Canadian Journal on Aging 2009;28(4):347-357.

10. Kessler RC, Andrews G, Colpe LJ, et al. Short screening scales to monitor population prevalences and trends in non-specific psychological distress. Psychological medicine 2002;32(6):959-976.

11. Spitzer RL, Kroenke K, Williams JB, et al. A brief measure for assessing generalized anxiety disorder: the GAD-7. Archives of internal medicine 2006;166(10):1092-1097.

12. Vézina J, Giroux L. L’Échelle des Embêtements: une étude de validation et d’adaptation du Hassles Scale pour une population adulte âgée. Paper presented at: meeting of the Canadian Psychological Association, Montreal, June1988.

13. Supina AL, Johnson JA, Patten SB, et al. The usefulness of the EQ-5D in differentiating among persons with major depressive episode and anxiety. Quality of Life Research 2007;16(5):749-754.

14. Helvik A-S, Corazzini K, Selbæk G, et al. Health-related quality of life in older depressed psychogeriatric patients: one year follow-up. BMC geriatrics 2016;16(1):131.

15. Hajek A, Brettschneider C, Ernst A, et al. Complex coevolution of depression and health-related quality of life in old age. Quality of Life Research 2015;24(11):2713-2722.

16. Hajek A, Brettschneider C, Lange C, et al. Gender differences in the effect of social support on health-related quality of life: results of a population-based prospective cohort study in old age in Germany. Qual Life Res 2016;25(5):1159-1168.

17. Diener E, Emmons RA, Larsen RJ, et al. The Satisfaction With Life Scale. J Pers Assess 1985;49(1):71-75.

18. Blais MR, Vallerand RJ, Pelletier LG, et al. L'échelle de satisfaction de vie: Validation canadienne-française du" Satisfaction with Life Scale.". Canadian Journal of Behavioural Science/Revue canadienne des sciences du comportement 1989;21(2):210.

19. Vasiliadis H-M, Latimer E, Dionne P-A, et al. The costs associated with antidepressant use in depression and anxiety in community-living older adults. The Canadian Journal of Psychiatry 2013;58(4):201-209.

20. Canadian Agency for Drugs and Technologies for Health (CADTH). Guidelines for the economic evaluation of health technologies: Canada. 4th ed. ed. Ottawa, Ontario: CADTH; 2017.

21. Canadian Agency for Drugs Technologies in Health. Guidelines for the economic evaluation of health technologies: Canada. In: *Guidelines for the economic evaluation of health technologies: Canada.* CADTH; 2006.

22. Reinharz D, Lesage AD, Contandriopoulos AP. Cost-effectiveness analysis of psychiatric deinstitutionalization. Canadian Journal of Psychiatry 2000;45(6):533-538.

23. Drummond MF, Sculpher MJ, Claxton K, et al. Methods for the economic evaluation of health care programmes. Oxford university press; 2015.

24. Ministère de la Santé et des Services Sociaux (MSSS). AS-471 – RAPPORTS FINANCIERS ANNUELS DES ÉTABLISSEMENTS. MSSS. <https://www.donneesquebec.ca/recherche/fr/dataset/as-471-rapports-financiers-annuels-des-etablissements/resource/ffc05092-520b-4383-ab87-292443a8cbd0>. Published 2018. Updated 2018-12-06. Accessed November 12, 2020.

25. Ministère de la Santé et des Services Sociaux (MSSS). Liste par centre d’activités MSSS. <http://msssa4.msss.gouv.qc.ca/fr/document/d26ngest.nsf/lca?OpenView>. Accessed.

26. Vasiliadis H-M, Dionne PA, Preville M, et al. The excess healthcare costs associated with depression and anxiety in elderly living in the community. American Journal of Geriatric Psychiatry 2013;21(6):536-548.

27. Rosenheck R, Frisman L, Neale M. Estimating the capital component of mental health care costs in the public sector. Administration and Policy in Mental Health and Mental Health Services Research 1994;21(6):493-509.

28. Government of Canada. Classes of depreciable property. Government of Canada. <https://www.canada.ca/en/revenue-agency/services/tax/businesses/topics/sole-proprietorships-partnerships/report-business-income-expenses/claiming-capital-cost-allowance/classes-depreciable-property.html>. Published 2020. Updated 2020-05-01. Accessed November 10th 2020.

29. Le Commissaire de la Santé et au Bien-Être. *Les urgences au Québec : Évolution de 2003-2004 À 2012-2013* Governement du Québec;2014.

30. Commission des normes dle, de la sante et de la securité du travail,. History of the minimum wage. <https://www.cnt.gouv.qc.ca/en/wages-pay-and-work/wages/history-of-the-minimum-wage/index.html>. . Published 2020. Accessed October 26, 2020.

31. Gamache P, Hamel D, Pampalon R. L’indice de défavorisation matérielle et sociale: en bref. In: INSPQ; 2015.

32. Statistics Canada. Canadian Community Health Survey (CCHS) - 2012. <https://www23.statcan.gc.ca/imdb-bmdi/instrument/3226_Q1_V9-eng.htm>. Published 2014. Accessed.

33. Bice TW, Boxerman SB. A Quantitative Measure of Continuity of Care. Medical Care 1977;15(4):347-349.

34. Duhoux A, Fournier L, Gauvin L, et al. Quality of care for major depression and its determinants: a multilevel analysis. BMC Psychiatry 2012;12:142.

35. Katzman MA, Bleau P, Blier P, et al. Canadian clinical practice guidelines for the management of anxiety, posttraumatic stress and obsessive-compulsive disorders. BMC psychiatry 2014;14(1):S1.

36. MacQueen GM, Frey BN, Ismail Z, et al. Canadian Network for Mood and Anxiety Treatments (CANMAT) 2016 clinical guidelines for the management of adults with major depressive disorder: section 6. special populations: youth, women, and the elderly. The Canadian Journal of Psychiatry 2016;61(9):588-603.

37. Parikh SV, Quilty LC, Ravitz P, et al. Canadian Network for Mood and Anxiety Treatments (CANMAT) 2016 clinical guidelines for the management of adults with major depressive disorder: section 2. Psychological treatments. The Canadian Journal of Psychiatry 2016;61(9):524-539.

38. Wolitzky-Taylor KB, Horowitz JD, Powers MB, et al. Psychological approaches in the treatment of specific phobias: A meta-analysis. Clinical psychology review 2008;28(6):1021-1037.

39. Roberge P, Fournier L, Duhoux A, et al. Mental health service use and treatment adequacy for anxiety disorders in Canada. Soc Psychiatry Psychiatr Epidemiol 2011;46(4):321-330.

40. Turgeon M, Guénette L. Portrait de l'usage des antidépresseurs chez les adultes assurés par le régime public d'assurance médicaments du Québec: rapport final. Conseil du médicament; 2011.

41. Canadian Coalition for Senior’s Mental Health. *National Guidelines for Seniors’ Mental Health. The Assessment and Treatment of Depression.* 2006.

42. Canadian Psychiatric Association. Clinical practice guidelines. Management of anxiety disorders. Canadian journal of psychiatry Revue canadienne de psychiatrie 2006;51(8 Suppl 2):9S.

43. Mireault J, Lemay A. Analyses cliniques des hospitalisations de la population de ville de Laval et des patients de la Cité de la Santé de Laval, Montréal. Association des hôpitaux du Québec 1999;113.

44. Statistics Canada. Enquête sur la santé dans les collectivités canadiennes (ESCC) - Santé mentale - Questionnaire. <http://www23.statcan.gc.ca/imdb-bmdi/instrument/5105_Q1_V3-fra.pdf>. Published 2013. Accessed March 3, 2021.
